# Effects of receptor for advanced glycation endproducts on microvessel formation in endometrial cancer

**DOI:** 10.1186/s12885-016-2126-3

**Published:** 2016-02-12

**Authors:** Lu Zheng, Da Li, Yi-Ming Zhou, Hui Yang, Di Cheng, Xiao-Xin Ma

**Affiliations:** Department of Obstetrics and Gynecology, Shengjing Hospital of China Medical University, Shenyang, 110004 China; Department of Obstetrics and Gynecology, Central Hospital of Shenyang Medical College, Shenyang, 110024 China; Department of Medicine, Brigham and Women’s Hospital, Harvard Institutes of Medicine, Harvard Medical School, Boston, MA 02115 USA

**Keywords:** RAGE, Microvascular, Proliferation, Endometrial cancer

## Abstract

**Background:**

The receptor for advanced glycation endproducts (RAGE) and microvascular status both play a critical role in cancer progression. However, the crosstalk between RAGE and microvascular formation in endometrial cancer remains largely unknown.

**Methods:**

RAGE expression and microvessel density were examined in 20 cases of normal endometrial tissue, 37 cases of well-differentiated endometrial cancer tissue, and 35 cases of poorly-differentiated endometrial cancer tissue. Regression analysis was used to examine the relationship between RAGE and microvessel density. The knockdown of RAGE was achieved using a small interfering RNA in HEC-1A endometrial cancer cells. A xenografted tumour model was used to evaluate RAGE-mediated microvascular formation and proliferation of endometrial cancer cells.

**Results:**

It was shown that (i) RAGE expression gradually increased in normal endometrium, well-differentiated endometrial cancer, and poorly-differentiated endometrial cancer, respectively; (ii) a positive correlation existed between RAGE and microvessel density in human endometrial cancer samples; (iii) RAGE knockdown was effective in decreasing microvessel formation in xenografted tumour models; and (iv) RAGE knockdown can significantly inhibit the proliferation of endometrial cancer cells in vivo.

**Conclusions:**

These results indicate that RAGE may be a potential trigger in microvascular formation and proliferation in the development of endometrial cancer.

**Electronic supplementary material:**

The online version of this article (doi:10.1186/s12885-016-2126-3) contains supplementary material, which is available to authorized users.

## Background

Endometrial cancer is the most common gynaecologic malignancy, and its incidence is increasing [1]. Accumulating evidence suggests that diabetes is a high risk factor for endometrial cancer [2], with an epidemiological study demonstrating an increased incidence of endometrial cancer in diabetic patients [3]. The receptor for advanced glycation endproducts (RAGE) was first identified as a signal receptor for advanced glycation endproducts (AGEs) [4], the products of non-enzymatic glycation/oxidation of proteins/lipids which have been linked to an increased risk of microvascular complications associated with diabetes [4, 5]. Interestingly, several studies have indicated that (i) microvessel density was significantly lower in renal cell carcinoma expressing low levels of RAGE [6]; (ii) RAGE was highly expressed in colorectal cancer tissues, and was associated with increased microvessel density [7]; (iii) blockade of ligand-RAGE interactions can prevent or delay diabetes-related structural microvessel complications in mice [8]; (iv) microvessel density may be a novel prognostic factor in various tumours [9, 10]. However, the role of RAGE and its related microvascular status in the pathogenesis of endometrial cancer remains largely unknown. In addition, although little is known to date on the direct role of RAGE in the proliferation of endometrial cancer, an emerging body of evidence suggests that RAGE plays an important role in promoting cell proliferation and survival in prostate cancer [11], lung cancer [12], breast cancer [13], and eukaemia cells [14]. Therefore, insights into the complex interrelationship among RAGE, microvascular formation and proliferation might improve current understanding of the basic molecular mechanisms of endometrial cancer.

## Methods

### Patients and tissue collection

This study was approved by the Institutional Review Board at China Medical University, and all subjects provided informed consent. Twenty cases of normal endometrial tissue (mean age, 55.6 ± 14.5), thirty-seven cases of well-differentiated endometrial cancer tissue (mean age, 58.4 ± 20.2), and thirty-five cases of poorly-differentiated endometrial cancer tissue (mean age, 60.3 ± 17.6) were collected from subjects at Shengjing Hospital of China Medical University between 2008 and 2012. Their characteristics are given in Additional file 1. All tissues were obtained during primary surgery, prior to administration of chemotherapy or radiotherapy, and were pathologically examined by haematoxylin and eosin staining. Endometrial cancer tissues were staged according to the International Federation of Gynecology and Obstetrics (FIGO 2009) by three experienced pathologists.

### Cell culture and transfection

Well-differentiated (Ishikawa) and poorly-differentiated endometrial cancer cell lines (HEC-1A) were maintained in DMEM supplemented with 10 % foetal bovine serum (Invitrogen, CA, USA). Cholesterol-conjugated small interfering RNA (siRNA) against RAGE was obtained from Biomics Biotechnologies (Shanghai, China), and synthesized as follows: 5′-GACCAACUCUCUCCUGUAUTT-3′ and 5′-AUACAGGAGAGAGUUGGUCTT-3′. A non-targeting siRNA duplex sequence was used as a negative control. The transfection protocol was as follows: 10 μl of siRNA was added to 175 μl of Opti-MEM (Invitrogen) reduced serum medium and mixed gently. At the same time, 4 μl of oligofectamin (Invitrogen) was added to 11 μl of Opti-MEM. After 5 min, Opti-MEM containing the siRNA was mixed gently with Opti-MEM containing oligofectamin. After 20 min, the mixture was added to the culture wells, and plates were incubated at 37 °C and 5 % CO_2_ for 24 h. Stable cell clones were identified by western blotting.

### Xenografted tumour model

All experiments were conducted according to the NIH Guide for Care and Use of Laboratory Animals, and all experimental procedures involving animals were approved by the Animal Care and Use Committee of China Medical University. Female BALB/c nude mice (5 weeks old) were purchased from the China Medical University Animal Centre. HEC-1A cells or RAGE-knockdown HEC-1A cells (1 × 10^7^ cells mixed with Matrigel in a final volume of 200 μl) were injected subcutaneously into the right armpit of BALB/c nude mice (n = 12 for each group). The growth of xenografted tumours was observed daily and the long and short diameters of tumours were recorded every 5 days; tumour volume was subsequently calculated by V (mm^3^) = (π × long diameter × short diameter^2^)/6. At 20 days, the animals were sacrificed and tumour tissues were collected. The expression of RAGE, Ki67 and CD34 in tumour tissues was determined by immunohistochemistry.

### Immunohistochemical analysis

Immunohistochemistry was performed as previously described [15]. The primary antibodies used were anti-RAGE (1:200; Santa Cruz Biotechnology, CA, USA), anti-CD34 (1:100; Dako, Glostrup, Denmark), and anti-Ki67 (1:100; Dako, Glostrup, Denmark). Microvessel density was assessed in anti-CD34-(for blood vessels) immunostained specimens. Area quantification was performed by light microscopy and analysed by Image-Pro Plus 6.0 (Media 2 Cybernetics, USA). Immunostaining was evaluated by two independent pathologists, blinded to the identity of the subject groups.

### Western blotting for RAGE

Western blotting was performed as previously described [16]. Briefly, 30 μg protein was separated on 10 % SDS polyacrylamide gels and transferred to polyvinyl difluoride membranes (Millipore, MA, USA). The membranes were blocked in TBS containing 0.1 % Tween-20 and 5 % non-fat dry milk for 30 min at room temperature, and incubated with antibody to RAGE (1:500; Santa Cruz Biotechnology) overnight at 4 °C. Then, membranes were washed with PBS-Tween followed by 1 h incubation at room temperature with horseradish peroxidase-conjugated secondary antibody (1:5000; Santa Cruz Biotechnology) and detected using enhanced chemiluminescence (Amersham Life Science, NJ, USA).

### Statistical analyses

Regression analysis was used to examine the relationship between RAGE and microvessel density. The data are presented as mean ± SD. Statistical differences in the data were evaluated by Student’s *t* test or one-way ANOVA as appropriate, and were considered significant at *P* < 0.05.

## Results

### Differences in RAGE expression patterns in endometrial cancer and endometrial cancer cell lines

**Fig. 1:**
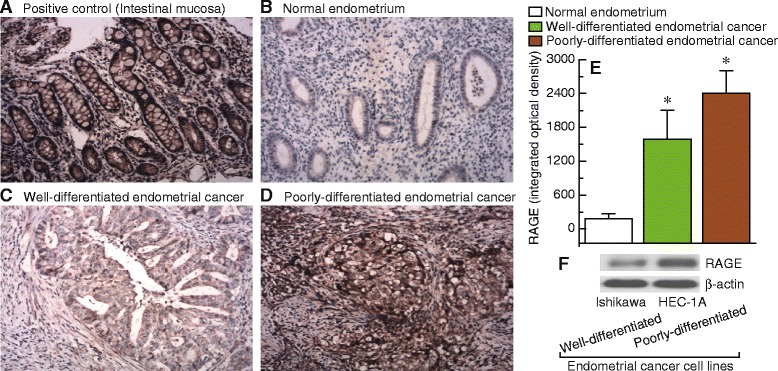
Expression patterns of RAGE in endometrial cancer and endometrial cancer cell lines. **a-d**, sections were subjected to immunostaining for RAGE in intestinal mucosa (positive control), normal endometrium, well-differentiated endometrial cancer, and poorly-differentiated endometrial cancer, respectively. Magnification is 100×. E, summary of the integrated optical densities of RAGE from the measurements taken in b-d. Bar graphs display mean ± SD. * *P* < 0.05 vs. normal. F, detection of RAGE protein levels in well-differentiated (Ishikawa) and poorly-differentiated (HEC-1A) endometrial cancer cell lines

Immunohistochemical analysis showed that the levels of RAGE expression gradually increased in normal endometrium, well-differentiated endometrial cancer, and poorly-differentiated endometrial cancer, respectively (Fig. 1a-e). It is interesting to note that poorly-differentiated endometrial cancer cell (HEC-1A) showed significantly increased expression of RAGE compared with well-differentiated endometrial cancer cells (Ishikawa) (Fig. 1f).

### RAGE expression correlated positively with microvessel density in endometrial cancer samples

**Fig. 2:**
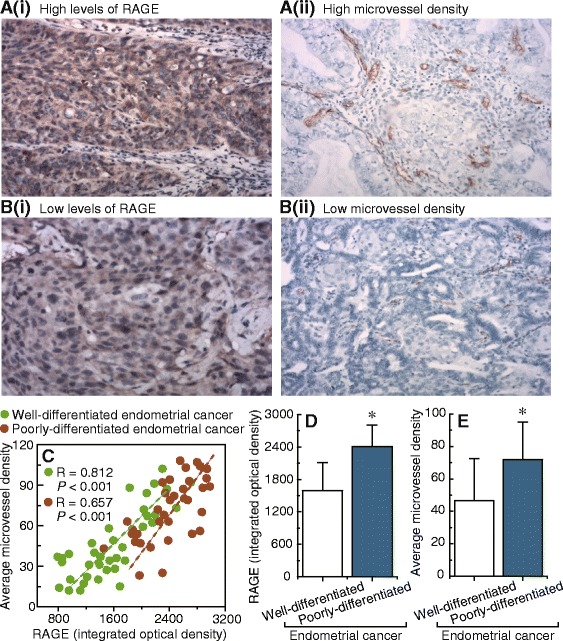
Correlation between RAGE expression and microvessel density in endometrial cancer samples. **a** and **b**, examples of immunostaining showing the positive correlation between the expression levels of RAGE and microvessel density in 72 endometrial cancer tissue samples. a(i) and a(ii), high RAGE levels and high microvessel density. b(i) and b(ii), low RAGE levels and low microvessel density. Magnification is 100× for a(i) and b(i), 200× for a(ii) and b(ii). **c**, correlation between RAGE expression and microvessel density in well-differentiated and poorly-differentiated endometrial cancer tissues, respectively. **d**, summary of the results obtained from the measurements shown in a(i) and b(i). E, summary of the results obtained from the measurements shown in a(ii) and b(ii). Bar graphs display mean ± SD. * *P* < 0.05 vs. control

Of particular interest and potential clinical relevance, the relationship between RAGE expression and microvessel density was studied in 72 human endometrial cancer specimens. Our results suggested that high levels of RAGE were associated with higher microvessel densities (Fig. 2a(i) and a(ii)), while low levels of RAGE were observed along with lower microvessel densities (Fig. 2b(i) and b(ii)) in endometrial cancer tissues. A significant positive association was shown to exist between RAGE expression and microvessel density in both well-differentiated (*R* = 0.812, *P* < 0.001) and poorly-differentiated endometrial cancer (*R* = 0.657, *P* < 0.001) (Fig. 2c). Poorly-differentiated endometrial cancer tissues consistently displayed higher levels of RAGE and microvessel density, compared with well-differentiated endometrial cancer tissues (Fig. 2d and e).

### RAGE can regulate microvessel formation in xenografted tumour models

**Fig. 3:**
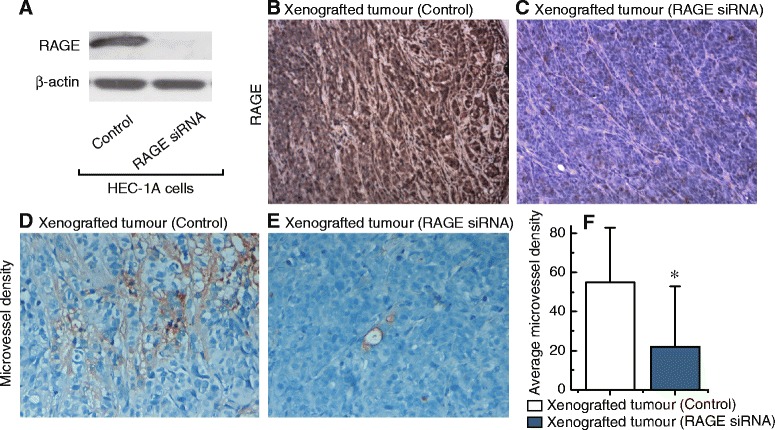
Effects of RAGE on microvessel density. **a**, western blot of RAGE expression before and after knockdown by siRNA in HEC-1A cells. **b** and **c**, sections subjected to immunostaining for RAGE in xenografted tumours of transfected control or RAGE-knockdown HEC-1A cells. Magnification is 100×. **d** and **e**, sections subjected to immunostaining for microvessel density in xenografted tumours of transfected control or RAGE-knockdown HEC-1A cells. Magnification is 400×. **f**, summary of microvessel density from the measurements shown in D and E. Each group, *n* = 12. Bar graphs display mean ± SD. * *P* < 0.05 vs. control

To further examine the role of RAGE in the regulation of microvessel formation, the effects of RAGE knockdown were evaluated in xenografted tumour models, HEC-1A cells, or RAGE-knockdown HEC-1A cells (Fig. 3a) were subcutaneously transplanted into nude mice. After 20 days, knockdown of RAGE (Fig. 3b and c) was shown to have effectively decreased microvessel density (Fig. 3d-f) in xenografted tumours.

### RAGE knockdown can significantly inhibit the proliferation of endometrial cancer cells in vivo

**Fig. 4:**
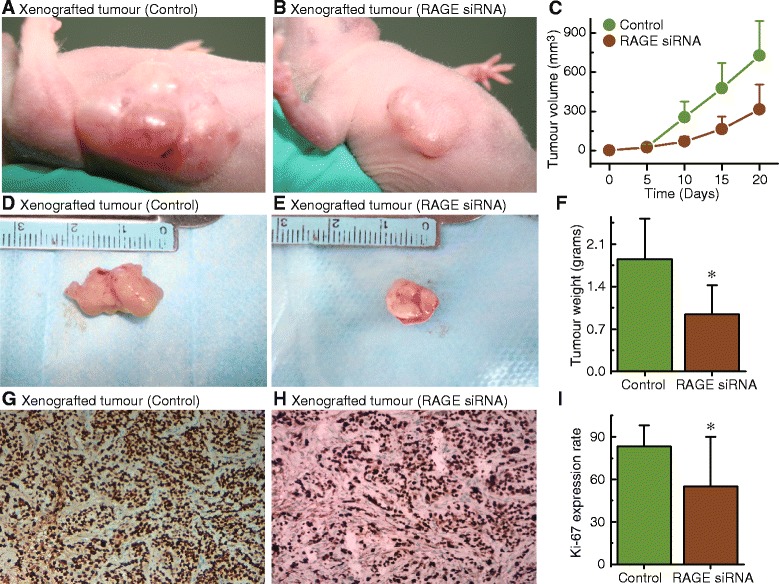
Effects of RAGE expression on xenografted tumour growth in nude mice. The xenografted tumour volume (**a** and **b**) and diameter (**d** and **e**) of nude mice transfected with control or RAGE-knockdown HEC-1A cells was shown. Magnification is 200×. **c**, summary of xenografted tumour volume at 0, 5, 10, 15, 20 days after transfection with HEC-1A cells (control) or RAGE-knockdown HEC-1A cells. F, xenografted tumour weight at 20 days in transfected control or RAGE-knockdown HEC-1A cells. **g** and **h**, sections were subjected to immunostaining for Ki67 in xenografted tumour with transfected control or RAGE-knockdown HEC-1A cells. Magnification is 200×. **i**, summary of the Ki67 levels from the measurements shown in g and h. Each group, *n* = 12. Bar graphs show mean ± SD. * *P* < 0.05 vs. control

Notably, we observed that the knockdown of RAGE significantly decreased xenografted tumour volume (Fig. 4a-c), diameter (Fig. 4d and e), and weight (Fig. 4f). In addition, the proliferation marker Ki-67 was also decreased after RAGE knockdown, suggesting that RAGE inhibition was an effective way to regulate endometrial cancer cell proliferation (Fig. 4g-i).

## Discussion

In this study, we report for the first time an association between RAGE and microvessel formation in endometrial cancer, as (i) RAGE expression was significantly higher in poorly-differentiated endometrial cancer compared with well-differentiated endometrial cancer and normal endometrial tissues; (ii) a positive correlation was shown to exist between RAGE expression and microvessel density in human endometrial cancer samples; (iii) RAGE knockdown was effective in decreasing microvessel density in xenografted tumour models; and (iv) Kaplan-Meier analysis and log-rank tests for overall survival revealed that RAGE levels showed a trend for poor overall survival (Additional file 2), but no significant difference was observed (*P* = 0.167). These results suggest that RAGE may act as a potential regulatory factor of microvessel formation in endometrial cancer, although a similar phenomenon has previously been observed in renal cell carcinoma [6] and colorectal cancer [7]. Folkman (1971) first proposed the concept of angiogenesis-dependent tumour growth [17], as once the original blood supply is exhausted, the tumour cannot grow without further blood supply [18, 19]. The evidence accumulated to date indicates a direct link between microvessel status and cancer development, and a growing body of data suggests that microvessel density is potentially involved in tumour recurrence, metastasis, and survival [9, 10, 20]. Therefore, the role of RAGE-mediated microvessel formation may provide new insights into the pathophysiology of endometrial cancer, although the precise nature of the regulatory mechanisms involved in this process requires further investigation. In addition, this study showed that RAGE may function as a key factor in the proliferation of endometrial cancer cells in vivo, although the molecular mechanisms are unclear, it is possible that (i) AGE/RAGE/PI3K/Akt signalling pathway-mediated Rb phosphorylation enhances prostate cancer cell proliferation [11]; (ii) the HMGB1-RAGE/TLR4-PI3K-Akt/Erk1/2 pathway contributes to the proliferation of lung cancer cells [12]; (iii) HMGB1-RAGE stimulates the phosphorylation of the JNK signalling pathway which promotes neural stem/progenitor cell proliferation [21]; (iv) RAGE inhibits osteoblast proliferation through the suppression of Wnt, PI3K, and ERK signalling [22]; (v) RAGE is involved in the proliferation of leukaemia cells via the MAPK, PI3K and JAK/STAT pathways [14]; and (vi) metformin inhibits AGEs-RAGE-mediated growth of MCF-7 breast cancer cells by the AMP-activated protein kinase pathway [13]. Some or all of these mechanisms may also be involved in RAGE-mediated proliferation of endometrial cancer cells.

## Conclusions

Our results indicate that RAGE may be a potential regulator of microvessel density and cell proliferation in endometrial cancer. Based on these findings, some interesting considerations for future studies can be identified; for example, how RAGE expression affects microvessel formation and the specific mechanism of RAGE-related endometrial cancer cell proliferation. The outcomes of this future work may improve our understanding of the basic molecular mechanisms behind RAGE-related endometrial cancer progression.

## Additional files

Additional file 1:
**Clinical characteristics for endometrial cancer patients.** (XLS 32 kb) Additional file 2:
**Kaplan-Meier analysis of overall survival for endometrial cancer patients.** (PDF 268 kb)

## Abbreviations

AGEs: advanced glycation endproducts
RAGE: receptor for advanced glycation endproducts
siRNA: small interfering RNA

## References

[1] Morice P, Leary A, Creutzberg C, Abu-Rustum N, Darai E. Endometrial cancer. Lancet. 2015;S0140-6736(15)00130-0. 10.1016/S0140-6736(15)00130-0 26354523

[2] Liao C, Zhang D, Mungo C, Tompkins DA, Zeidan AM (2014). Is diabetes mellitus associated with increased incidence and disease-specific mortality in endometrial cancer? A systematic review and meta-analysis of cohort studies. Gynecol Oncol.

[3] Shikata K, Ninomiya T, Kiyohara Y (2013). Diabetes mellitus and cancer risk: review of the epidemiological evidence. Cancer Sci.

[4] Ramasamy R, Yan SF, Schmidt AM (2011). Receptor for AGE (RAGE): signaling mechanisms in the pathogenesis of diabetes and its complications. Ann N Y Acad Sci.

[5] Stirban A, Gawlowski T, Roden M (2014). Vascular effects of advanced glycation endproducts: Clinical effects and molecular mechanisms. Mol Metab.

[6] Guo Y, Xia P, Zheng JJ, Sun XB, Pan XD, Zhang X (2015). Receptors for advanced glycation end products (RAGE) is associated with microvessel density and is a prognostic biomarker for clear cell renal cell carcinoma. Biomed Pharmacother.

[7] Liang H, Zhong Y, Zhou S, Peng L (2011). Knockdown of RAGE expression inhibits colorectal cancer cell invasion and suppresses angiogenesis in vitro and in vivo. Cancer Lett.

[8] Kim W, Hudson BI, Moser B, Guo J, Rong LL, Lu Y (2005). Receptor for advanced glycation end products and its ligands: a journey from the complications of diabetes to its pathogenesis. Ann N Y Acad Sci.

[9] Kelly T, Miao HQ, Yang Y, Navarro E, Kussie P, Huang Y (2003). High heparanase activity in multiple myeloma is associated with elevated microvessel density. Cancer Res.

[10] Sato M, Nakai Y, Nakata W, Yoshida T, Hatano K, Kawashima A (2014). Microvessel area of immature vessels is a prognostic factor in renal cell carcinoma. Int J Urol.

[11] Bao JM, He MY, Liu YW, Lu YJ, Hong YQ, Luo HH (2015). AGE/RAGE/Akt pathway contributes to prostate cancer cell proliferation by promoting Rb phosphorylation and degradation. Am J Cancer Res.

[12] Xu X, Zhu H, Wang T, Sun Y, Ni P, Liu Y (2014). Exogenous high-mobility group box 1 inhibits apoptosis and promotes the proliferation of lewis cells via RAGE/TLR4-dependent signal pathways. Scand J Immunol.

[13] Ishibashi Y, Matsui T, Takeuchi M, Yamagishi S (2013). Metformin inhibits advanced glycation end products (AGEs)-induced growth and VEGF expression in MCF-7 breast cancer cells by suppressing AGEs receptor expression via AMP-activated protein kinase. Horm Metab Res.

[14] Kim JY, Park HK, Yoon JS, Kim SJ, Kim ES, Ahn KS (2008). Advanced glycation end product (AGE)-induced proliferation of HEL cells via receptor for AGE-related signal pathways. Int J Oncol.

[15] Li D, Bi FF, Cao JM, Cao C, Li CY, Yang Q (2013). Effect of BRCA1 on epidermal growth factor receptor in ovarian cancer. J Exp Clin Cancer Res.

[16] Li D, Bi FF, Chen NN, Cao JM, Sun WP, Zhou YM (2014). A novel crosstalk between BRCA1 and sirtuin 1 in ovarian cancer. Sci Rep.

[17] Folkman J (1995). Seminars in Medicine of the Beth Israel Hospital, Boston. Clinical applications of research on angiogenesis. N Engl J Med.

[18] Kukreja I, Kapoor P, Deshmukh R, Kulkarni V (2013). VEGF and CD 34: A correlation between tumor angiogenesis and microvessel density-an immunohistochemical study. J Oral Maxillofac Pathol.

[19] Li JY, Zhang Y, Zhang WH, Jia S, Kang Y, Tian R (2013). Effects of differential distribution of microvessel density, possibly regulated by miR-374a, on breast cancer prognosis. Asian Pac J Cancer Prev.

[20] Chen Y, Yan J, Yuan Z, Yu S, Yang C, Wang Z (2013). A meta-analysis of the relationship between lymphatic microvessel density and clinicopathological parameters in breast cancer. Bull Cancer.

[21] Li M, Sun L, Luo Y, Xie C, Pang Y, Li Y (2014). High-mobility group box 1 released from astrocytes promotes the proliferation of cultured neural stem/progenitor cells. Int J Mol Med.

[22] Li G, Xu J, Li Z (2012). Receptor for advanced glycation end products inhibits proliferation in osteoblast through suppression of Wnt, PI3K and ERK signaling. Biochem Biophys Res Commun.

