# Supplementary figures and images for: Effects of receptor for advanced glycation endproducts on microvessel formation in endometrial cancer
Source: BMC Cancer. 2016 Feb 12;16:93. doi: 10.1186/s12885-016-2126-3 (PMC4751660; doi:10.1186/s12885-016-2126-3)

Kaplan-Meier analysis of overall survival for endometrial cancer patients

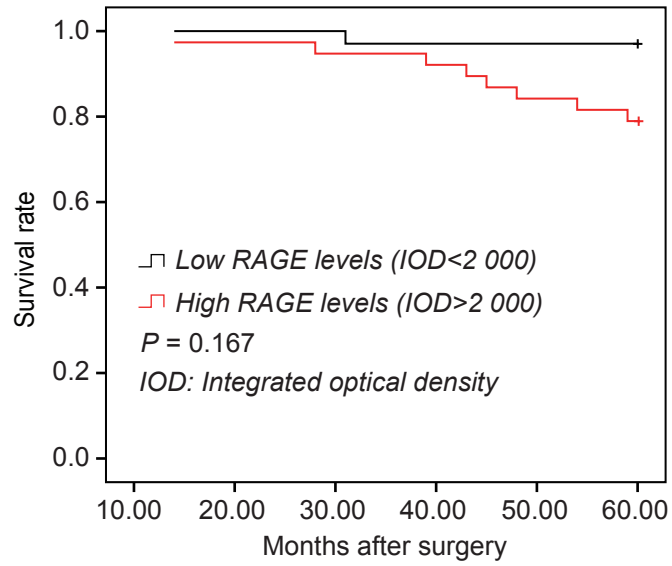

Supplement: Additional file 2: — Kaplan-Meier analysis of overall survival for endometrial cancer patients. (PDF 268 kb) [file 12885_2016_2126_MOESM2_ESM.pdf]
